# Impact of short stature on health-related quality of life in long-term survivors of acute lymphoblastic leukemia in childhood and adolescence

**DOI:** 10.1186/s41687-018-0084-z

**Published:** 2018-12-07

**Authors:** Laura Collins, Uma Athale, Amy Cranston, Ronald Barr

**Affiliations:** 1 McMaster Children’s Hospital, Hamilton Health Sciences, Hamilton, Ontario Canada; 2 Department of Pediatrics, McMaster University, Room 3N27, Health Sciences Centre, 1200 Main Street West, Hamilton, Ontario L8S 4J9 Canada

**Keywords:** Short stature, Health-related quality of life, Survivors, Acute lymphoblastic leukemia

## Abstract

**Purpose:**

Some survivors of acute lymphoblastic leukemia (ALL) in childhood and adolescence exhibit short stature, especially if their treatment included cranial irradiation. The impact of this outcome on health-related quality of life (HRQL) is uncertain and so formed the basis for the investigation reported here.

**Methods:**

This study examined the association between self-reported HRQL and measured height in a cohort (*n* = 75) of survivors of ALL more than 10 years from diagnosis. HRQL was expressed as utility scores generated from a multi-attribute preference-based measure, the Health Utilities Index (HUI) which includes the complementary systems HUI2 and HUI3. For single attributes the range is from 1.00 (no limitations) to 0.00 (lowest level of function). For overall HRQL the range is 1.00 (perfect health) to 0.00 (equivalent to being dead). A negative score is associated with states of health worse than being dead.

**Results:**

There were no statistically significant differences in overall HRQL between subjects <25th (*n* = 16, 21%), 15th (*n* = 11, 15%) and 10th (*n* = 10, 13%) centiles. A greater amount of emotional morbidity, focused on anger and depression, was manifest in those <25th and 15th centiles, with clinically important differences of 0.07 (*p* = 0.03) and 0.077 (*p* = 0.016) respectively, but not in the shortest group who were < 10th centile.

**Conclusions:**

Studies in large cohorts of young adults in the general population has reported an inconsistent relationship between height and HRQL. Results from the current study suggest that no such relationship exists in long-term survivors of ALL in childhood and adolescence.

## Introduction

Among the late sequelae of the treatment of acute lymphoblastic leukemia (ALL) in children are diminished final height (short stature) and overweight/obesity [1]. In 2007 colleagues in Brazil [2] identified a considerable literature on short stature in such populations while presenting their own results which revealed that the adverse effect on final height began during treatment and was especially evident in those who had undergone cranial radiotherapy (CRT). Also in 2007 the Childhood Cancer Survivor Study (CCSS) described the results of a cross-sectional evaluation in which survivors of ALL self-reported their heights [3]. Even those who had received chemotherapy only were shorter than their siblings on average, though only 1.9% of this sub-group had Z scores for height of less than − 2.0. The CCSS investigators observed also that another sub-group, who had undergone total body irradiation (TBI), were not only much shorter but often manifest sarcopenic obesity. A more recent CCSS study reported on almost 3500 long term survivors of acute leukemia, most with ALL, at a median follow-up of more than 20 years [4]. A small group had received TBI. Short stature was defined as a height Z score less than − 1.96. In those who received no CRT the prevalence was 3.8% while in those who had had CRT but not TBI 10.1% were of short stature.

Despite this abundance of information on diminished final height in survivors of ALL there are very limited data on the broader impact of this outcome, although the association of short stature with psychological morbidity in children at large has been well recognised for at least 25 years [5]. Yet we are aware of only one report of measuring health-related quality of life (HRQL) in survivors of cancer in childhood (including a group who had had ALL) that focused on growth [6]. This study examined the influence of growth hormone and the relationship of HRQL to stature was not presented.

Against this background we undertook a study of HRQL in relation to stature among long-term survivors of ALL who were subjects in a comprehensive examination of body composition and bone health [7]. Within this cohort HRQL was lower in those who had sarcopenic obesity, a prevalent outcome in these survivors [8].

## Methods

Details of the comprehensive study have been published [7]. A sample of 75 subjects was assembled from a cohort of children and adolescents who had been diagnosed with ALL at McMaster Children’s Hospital at least a decade earlier. All had been treated on Dana Farber Cancer Institute Childhood ALL Consortium protocols [9]. Almost all subjects are Caucasian and they were 13.5–38.5, median 21.2, years of age at the time of the study. The subjects were 10–26, median 15, years from diagnosis.

Subjects aged 20 and older were assumed to have reached their final height. Percentiles and Z scores for height were determined by WHO criteria [10] that range from birth to 19 years 11 months.

HRQL was measured by the Health Utilities Index (HUI) which is a family of multi-attribute, preference-based instruments developed at McMaster University [11]. HUI2 and HUI3 are complementary systems that provide utility scores for individual attributes (domains or dimensions) of health as well as multi-attribute overall HRQL (the comprehensive health state) [12]. The attributes in HUI2 are Sensation, Mobility, Self-Care, Cognition, Emotion, Pain and Fertility. In HUI3 the attributes are Vision, Hearing, Speech, Ambulation, Dexterity, Cognition, Emotion and Pain. The constructs for Cognition, Emotion and Pain are different in HUI2 and HUI3. Cognition in HUI2 addresses learning and remembering; in HUI3 memory/forgetfulness and solving day-to-day problems. Emotion in HUI2 addresses anger and depression; in HUI3 happiness and unhappiness. Pain in HUI2 addresses use of analgesics; in HUI3 limitation of normal activities. Single attribute scores range from 1.00 (no limitations) to 0.00 (the lowest level of function) and overall HRQL scores range from 1.00 (perfect health) to 0.00 (equivalent to being dead). Clinically meaningful differences (CIDs) in utility scores are ≥0.05 for single attributes [13] and ≥ 0.03 for overall HRQL [14]. The HRQL data were taken from a recent report on the same cohort [15].

Sarcopenic obesity was defined as before – a combination of a negative Z score for appendicular lean mass index (ALM/height^2^) with a positive Z score for fat mass index (FM/height^2^)^8^.

Descriptive statistics, namely mean (with standard deviation [SD] of the mean) and median (with minimum and maximum values) were used to report continuous variables and numbers (percent) for categorical variables. In order to answer the study question “do participants with short stature have poorer HRQL?”, using univariate analyses we compared the overall HRQL and single attribute utility scores between subjects with and without short stature, defined as height less than or equal to the 25th percentile. We performed additional analyses using the height threshold of the 15th percentile. Given the limited sample size multivariable analyses were not possible. The limit of statistical significance was set at alpha = 0.05 and 95% confidence intervals were reported for measures of association for all comparisons. Statistical analyses were performed with SPSS (version 24).

## Results

Among the 75 subjects there were 39 males including 12 men ages 20–24. Fifty-one subjects had received CRT; none had received TBI and none had undergone hematopoietic stem cell transplantation. No one had been given growth hormone. The height centiles and Z scores are displayed in Table 1. Ten subjects were < 10th centile; 7 were female; 8 had been irradiated, including all 7 females. Three subjects were < 5th centile; all were female and 2 had been irradiated. Six subjects had Z scores <− 1.50; all were female and 5 had been irradiated. The mean Z scores for females were − 0.699 and + 0.216 in irradiated and non-irradiated subjects respectively (*p* = 0.009). There was no corresponding difference in males. Five subjects are receiving thyroid hormone replacement (3 females and 2 males) and 4 had CRT. In this small sub-group the mean height percentile was 39 (median 38, range 16 to 57) and the mean height Z score was - 0.29 (median − 0.30, range - 0.96 to 0.19).

**Table 1:** Height percentiles and Z scores according to CRT^a^ and sarcopenia obesity (SO)

	All subjects	CRT	No CRT	SO	No SO
N	75	51	24	31	42
Percentile					
Median	48	47	53	47	48.5
Range	1–96	1–96	2–93	2–96	1–93
Mean	41	41	54	44	48
SD^b^	29	27	27		
Z Score					
Median	−0.035	−0.070	0.360	−0.070	−0.025
Range	−2.32 to 1.87	−2.32 to 1.87	−2.04 to 1.55	−2.04 to 1.87	− 2.32 to 1.55
Mean	−0.120	−0.020	0.090	−0.192	− 0.079

^a^ *CRT* Cranial Radiotherapy

^b^ *SD* Standard Deviation

The height centile and Z scores for subjects with (*n* = 31) and without (*n* = 42) sarcopenic obesity are displayed in Table 1. There were no statistically significant differences in stature between the groups.

Overall HRQL scores by height are shown in Table 2 using both the 25th and 15th centiles to define short stature. In a comparison of those below and at/above the 25th centile there were no statistically significant or clinically important differences. While there were no statistically significant differences using the 15th centile the differences between those below and at/above this cut off were CIDs (0.07 for HUI2 and 0.11 for HUI3).

**Table 2:** Single attributes and overall health-related quality of life (HRQL) according to height percentiles

	< 25th	≥ 25th	< 15th	≥ 15th
HU12^a^	n = 16	*n* = 55	n = 11	*n* = 60
Cognition				
Mean (SD)^b^	0.96 (0.06)	0.93 (0.10)	0.96 (0.07)	0.93 (0.10)
Emotion				
Mean (SD)	0.96 (0.06)	0.89 (0.21)	0.97 (0.06)	0.90 (0.20)
Pain				
Mean (SD)	0.98 (0.02)	0.95 (0.15)	0.97 (0.02)	0.95 (0.14)
Overall HRQL				
Mean (SD)	0.86 (0.20)	0.89 (0.16)	0.82 (0.23)	0.89 (0.15)
HU13^c^	*n* = 18	n = 55	n = 13	n = 60
Cognition				
Mean (SD)^b^	0.95 (0.10)	0.91 (0.18)	0.94 (0.11)	0.91 (0.18)
Emotion				
Mean (SD)	0.98 (0.03)	0.93 (0.17)	0.98 (0.04)	0.94 (0.16)
Pain				
Mean (SD)	0.97 (0.06)	0.93 (0.17)	0.97 (0.07)	0.93 (0.17)
Overall HRQL				
Mean (SD)	0.81 (0.26)	0.83 (0.24)	0.73 (0.29)	0.84 (0.23)

^a^ Health Utilities Index Mark 2

^b^ Standard Deviation

^c^ Health Utilities Index Mark 3

Attention then turned to analysis of each attribute in HUI2 and HUI3 (Table 2). Comparing those <25th percentile for height with those who were taller the only CID in HUI2 was in Emotion (0.07) and it was the only difference that was statistically significant (*p* = 0.03). In HUI3 there were no differences that were clinically important, except for Emotion (0.051), or statistically significant. Using the cut off of the 15th centile, Emotion was again singled out in HUI2 as a CID (0.077) and statistically significant (*p* = 0.016). In HUI3 Emotion was neither a CID nor statistically significant.

In those subjects (*n* = 9) who had the lowest overall HRQL scores (≤ 0.50 in HUI3), the M:F ratio was 7:2 and 6 of 9 had received CRT (67% compared to 69% in the entire cohort). Height centiles ranged from 13 to 96 (median 48, mean 50) and only 1 was <25th. Height Z scores were − 0.57 to 1.87 (median − 0.04, mean 0.07) and only 1 was < − 1.00. The HUI2 scores were 0.23–0.64 (median 0.50, mean 0.49) and the HUI3 scores − 0.09 to 0.47 (median 0.31, mean 0.27). Examination of the single attribute utility scores for Cognition, Emotion and Pain produced the following. HUI2 Cognition 0.66–1.00 (median 1.00, mean 0.94); Emotion 0.00–1.00 (median 0.86, mean 0.71); Pain 0.00–1.00 (median 0.75, mean 0.76). HUI3 Cognition 0.32–1.00 (median 0.70, mean 0.63); Emotion 0.00–1.00 (median 0.91, mean 0.72); Pain 0.00–1.00 (median 0.92, mean 0.71).

Among the subjects (*n* = 10) who were shortest (height < 10th centile), the M:F ratio was 3:7 and 9 had received CRT. Height centiles were 1–8 (median 6, mean 5). Height Z scores were − 2.32 to − 1.37 (median − 1.54, mean − 1.66). HUI2 scores ranged from 0.87–1.00 (median 0.96, mean 0.95) and the HUI3 scores 0.63–1.00 (median 0.92, mean 0.88). Examination of the single attribute utility scores for Cognition, Emotion and Pain produced the following. HUI2 Cognition 0.95–1.00 (median 1.00, mean 0.99); Emotion 0.86–1.00 (median 1.00, mean 0.96); Pain 0.95–1.00 (median 1.00, mean 0.98). HUI3 Cognition 0.70–1.00 (median 1.00, mean 0.93); Emotion 0.91–1.00 (median 1.00, mean 0.98); Pain 0.77–1.00 (median 1.00, mean 0.96). In neither this nor the preceding analysis was there evidence of a “tale within the tail” [15].

## Discussion

Studies in children have defined idiopathic short stature as height less than the 5th centile for age and sex in the absence of illness, hormonal deficiency or a recognisable syndrome [16]. In a literature review in 2011 [17] Dr. Monika Bullinger concluded that it “did not provide conclusive evidence for differences in psychological status between short children and children of normal height in the general population”. However, in the same year, investigators in Texas, using the child self-report version of the health profile Peds QL™, reported that children being investigated for short stature had significant impairment of overall HRQL as well as in the physical, psychosocial, emotional and social domains compared to healthy children of normal height [16]. Interest in this area has increased sufficiently that a validated questionnaire has been developed by Bullinger and colleagues for use in 7 languages in Europe, including the UK [18], as well as in American English [19]. In a cross-cultural analysis using this instrument, the highest number of statements from patients and parents related to social and emotional needs and concerns [20].

Beyond childhood there is less information on the relationship between height and HRQL in the general population. In a study of 6646 adolescents aged 11–17 years in Germany, height was shown to be a weak predictor of HRQL assessed by the health profile KINDL-R [21], although those with “marginal emotional/behavioral problems” had significantly lower HRQL scores than those who did not. Two contrasting studies have been reported on large samples of adults in the general population. In the UK 14,416 subjects older than 18 years of age were assessed using the preference-based instrument EQ-5D for the measurement of HRQL [22]. There was a progressive decline in the overall score within the height range > + 2.5 to <− 3.0 SDS and a marked reduction in HRQL at SDS (Z) scores <− 2.0, equivalent to the 5th centile. However, a subsequent report from France, on 18,105 subjects aged 18–50 years, found that height was a very weak predictor of HRQL measured by the health profile SF-36 [23].

The subjects in our study were on average 15 years from diagnosis and almost 70% had received CRT, reflective of the era in which they were treated. While the average height in the irradiated group was lower than in the non-irradiated subjects, the proportions of the entire study sample less than the 25th and 15th centiles, 21.3 and 14.7% respectively, were unremarkable. Nevertheless, there was an ‘excess’ of subjects below the 10th and 5th centiles and the great majority of them had been irradiated.

Limitations of this study include the size of the cohort and the cross-sectional design. The proportion of subjects who had received CRT is much higher than in children undergoing current therapy but does allow for an analysis of the impact of this treatment modality.

In the earlier study of HRQL in this cohort, the most prevalent morbidity was in the attribute of Emotion in HUI2 [15]. Moreover, “the tale is in the tail” often with respect to HRQL [15] and one study subject had an HUI3 score for overall HRQL of − 0.09. Negative scores are associated with states of health worse than being dead [12]. Examination of the 9 subjects who had profoundly low overall HRQL revealed a combined burden of morbidity in the attributes of Cognition, Emotion and Pain (in both HUI2 and HUI3); those affected most frequently in survivors of ALL [24]. However, this did not appear to be associated with either short stature or CRT. Support for this lack of association is provided by the analysis of HRQL in the sub-group of subjects who were particularly short (< 10th centile). They had remarkably little morbidity in Cognition, Emotion and Pain. In summary, the results of this study suggest that short stature in long-term survivors of ALL is not associated with worse HRQL, perhaps because a height deficit in this population is a minor consideration in the context of other late effects of treatment.
